# Comparative Analyses of Soil Bacterial Colonies of Two Types of Chinese Ginger after a Major Flood Disaster

**DOI:** 10.1128/spectrum.04355-22

**Published:** 2023-02-06

**Authors:** Xinyang Li, Xiaokang Li, Jun Hong, Yan Wang, Duanqiang Guo, Jinlong Liu, Zewen Zhang, Wenwei He, Kaisheng Xue, Qingqing Wang

**Affiliations:** a Henan University of Urban Construction, Ping Dingshan, China; b Wuhan Jinxin Gynecology and Obstetrics Hospital of Integrative Medicine, Wuhan, China; Agroscope

**Keywords:** ginger, soil bacterial colony, diversity analysis, flooding disaster

## Abstract

Ginger, an important cash crop, has been cultivated for thousands of years in China. However, comparative studies on soil bacterial communities of Chinese ginger varieties, especially after flooding, are lacking. Here, we comprehensively compared the bacterial communities of two types of ginger soils from four different locations. Surprisingly, the 100-year flood (20 July 2021, in Henan, China) did not significantly affect the soil bacterial composition compared with previous reports. In contrast, flooding may have brought in nutrients and promoted the propagation of eutrophic bacteria, and *Alphaproteobacteria* were the most abundant in the Zhangliang region (~25%). However, due to the most severe flooding and inundation, the Zhangliang region, also probably contaminated with polycyclic aromatic hydrocarbons and heavy metals, showed the lowest microbial diversity. Moreover, the geographical location influenced the microbial communities more than did the soil type or ginger variety. These findings help us understand the species and composition of bacteria and infection of ginger after flooding and soaking. Further, the interaction mechanisms underlying these emerging phenomena need to be further investigated.

**IMPORTANCE** There are few comparative studies on the soil bacterial communities of Chinese ginger varieties after flooding. After a 100-year flood (20 July 2021, in Henan, China), we comprehensively compared the bacterial communities of two types of ginger soils from four different locations. Surprisingly, this flood did not significantly affect the soil bacterial composition compared with previous reports. In contrast, it was found that the flooding may have brought in nutrients and promoted the propagation of eutrophic bacteria for the Zhangliang region. However, the flooding had also brought in polycyclic aromatic hydrocarbon and heavy metal contamination. Moreover, we also verified that geographical location influenced the microbial communities more than did the soil type or ginger variety. These findings help us understand the species and composition of bacteria and infection of ginger after flooding and soaking.

## INTRODUCTION

Ginger (Zingiber officinale) has a deep historical background of extensive cultivation worldwide, especially in China, with more than 2,500 years of cultivation since the Spring and Autumn period of China (1, 2). Huaijiang (HJ) ginger, Zhangliang ginger, Panghai (PH) ginger, and Shannong ginger are some of the well-known Chinese ginger varieties. Ginger in Chinese medicines has been used to dispel cold, dispel sweat, promote hair growth, and strengthen the stomach (3, 4). In addition, ginger is an important flavor seasoning for Chinese cuisine, with the role of removing fishy fragrance, showing its great economic and medicinal value (3, 4). The growth and development of the ginger plant require several nutrients (including nitrogen, phosphorus, and potassium), which are circulated by microorganisms as decomposers (3, 5). However, along with beneficial microorganisms, soil also comprises some pathogenic microorganisms, which inhibit crop growth (3, 6). Thus, it is important to characterize the community structure of ginger soil microorganisms (2, 5).

In soil microbial sequencing, a bacterial genome’s 16S rRNA gene fragment is first amplified and sequenced, followed by studying the colony type and microorganism structure through bioinformatic analyses (3, 5, 7, 8). Recently, with the development of research and technology, second-generation high-throughput sequencing (NGS) technology has emerged, which is a modification of the traditional Sanger sequencing (9–11). This technology can sequence many microbial DNA molecules in the V4 region of a soil sample in parallel in a single run and has a substantial quantitative capability, where the number of times a DNA sample is sequenced directly reflects the DNA abundance of that sample. The analysis is usually performed using 16S rRNA sequencing and bioinformatics. Based on the sequencing results, the microbial communities are classified by the degree of similarity into operational taxonomic units (OTUs) (3, 7, 12). Based on OTUs, researchers can perform various soil microbial characterizations, such as soil microbial analyses of localized homogeneous environmental species diversity (α-diversity analysis), principal-component analysis (PCA), and composition analyses of microbial communities (3, 13).

**FIG 1:**
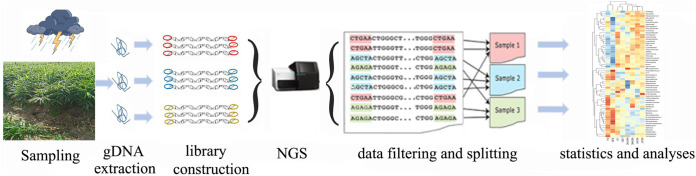
The general technical flow. The technical process consists of the following six steps: sampling, genome DNA extraction, library construction, NGS, data processing, and statistics and analyses.

Previous studies have comparatively analyzed the microbial communities of healthy and diseased ginger (3, 7). Also, turmeric and ginger intercropping affects the proportion of dominant bacteria and increases the active ingredient contents in patchouli (14). However, fewer studies have focused on the community structure of ginger soil bacteria after the floods of 20 July 2021, a 100-year flood disaster in Henan Province in north-central China. Many cash crops, including ginger, suffered from flooding and rain inundation for 2 or more months (15). We collected ginger soil samples in September/October 2021, when the floods had largely subsided. We investigated the effects of flood inundation on the diversity of bacterial colonies in ginger soil for 2 months. Our objective is to compare bacterial rhizosphere community structures of two types of ginger soils from four different locations subjected to a major flood disaster. The general technical flow is shown in Fig. 1.

## RESULTS

### Data processing and OTU acquisition.

After format conversion and sequencing data splitting, we extracted 4,365,450 OTU tags, averaging 136,420.31 OTU tags per group. Tags are the data showing that the filtered sequences correctly overlap and contain the correct barcode and high-quality reads. We obtained 4,198,089 clean OTU tags after low-quality data filtering, with a validity rate of 96.17% and an average of 1,311,190.28 clean OTU tags per group (see Fig. S1 in the supplemental material). For the following analyses, clean OTU data were used. To facilitate comparative analyses, four groups were assigned corresponding to the soil collection locations (Table 1).

**TABLE 1:** Four categorization methods of ginger soil^*a*^

Soil no.	Name	Category 1	Category 2	Category 3	Category 4
1	ZLBC01-04	ZLBC	Zhangliang or ZL		
2	ZLJ01-04	ZLJ	Zhangliang or ZL	ZLJ	ZLJ/LZJ or Panghai (PH) ginger
3	LZJ01-04	LZJ	Liuzhai or LZ	LZJ	ZLJ/LZJ or Panghai (PH) ginger
4	LZBC01-04	LZBC	Liuzhai or LZ		
5	MZSHBC01-04	MZSHBC	MZSH		
6	MZJSH01-04	MZJSH	MZSH	MZJSH	MZJ or Huaijiang (HJ) ginger
7	MZJST01-04	MZJST	MZST	MZJST	MZJ or Huaijiang (HJ) ginger
8	MZSTBC01-04	MZSTBC	MZST		

a We collected two types of ginger, PH and HJ. Four ginger soil groups were set up with their blank controls (BC), each group containing four samples, totaling 32 samples (Table 2). They were named ZLJ01-04, ZLBC01-04, MZJST01-04, MZSTBC01-04, MZJSH01-04, MZSHBC01-04, LZJ01-04, and LZBC01-04. The nomenclature rules were as follows: ZL, MZ, and LZ refer to the sampling site Lushan (Zhangliang township), Mengzhou (Gudan township), and Xinmi (Liuzhai township), respectively. J refers to the Chinese “jiang” (ginger) abbreviation. ST refers to sandy soil; SH refers to the combination of sandy and dark loessal soil.

**FIG 2:**
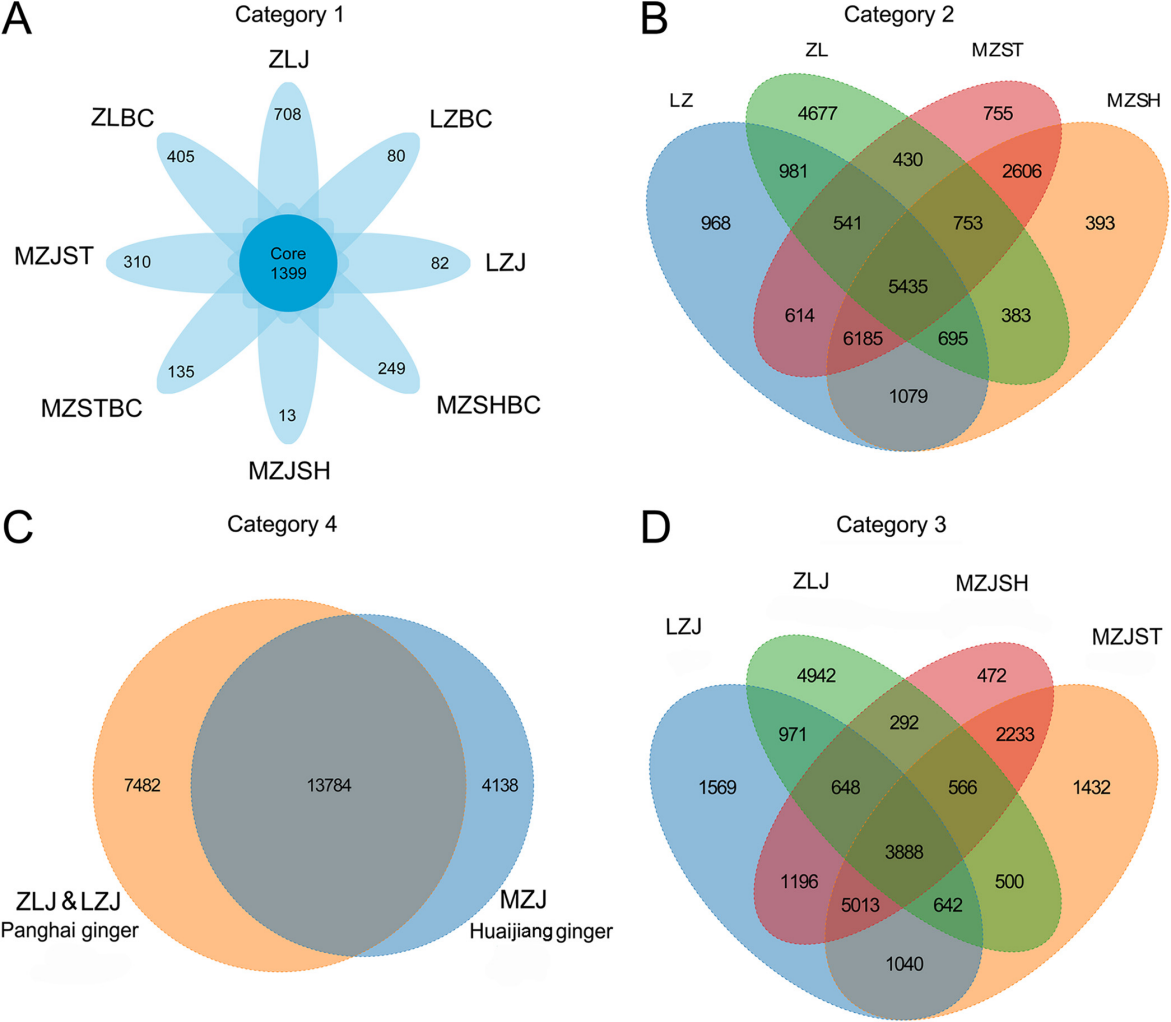
Identification of the shared and unique OTUs among groups using Venn diagrams. Four different classification methods are presented. (A) The first classification method was based on ginger groups and blank controls. (B) The second classification method was based on regions. (C) The third classification method was based on the four ginger groups. (D) The fourth classification method was based on ginger varieties (PH and HJ). The naming rules are as follows. ZL, MZ, and LZ refer to the sampling sites: Lushan (Zhangliang) and Mengzhou and Xinmi (Liuzhai). J refers to the abbreviation of Chinese “jiang” (ginger). ST refers to sandy soil; SH refers to the mixed soil of sandy soil and dark loessal soil. In addition, BC refers to “blank control.”

To identify the sharing and uniqueness of OTUs among groups, we plotted the Venn diagrams under different categorization method conditions (Fig. 2). In the first categorization method (category 1, Fig. 2A), each group, including the control group, had an amount of unique OTUs (13 to 708 species), with ginger soil in the MZJSH group having 13 unique OTUs (the least) and the ginger soil in the ZLJ group having 708 unique OTUs (the most). The number of these unique OTUs (bacterial communities) may be related to soil fertility and geographical location, and the type of these unique OTUs is likely related to the characteristic quality of the local ginger. However, in this study, the factor of flooding cannot be ignored, especially in the most severely affected regions of Zhangliang. The eight groups also shared 1,399 OTUs.

The second categorization method (category 2, Fig. 2B) and the third categorization method (category 3, Fig. 2D) were classified based on region (including the blank control groups) and ginger origin, respectively. In both categorization methods, the shared OTUs were similar, and any of the two groups had shared OTUs. Still, each group had its own unique OTUs. Although the soil types of MZST and MZSH were different, the two groups had the most OTUs in common, probably because their sampling locations are only 2 to 3 km apart. The fourth categorization method (category 4, Fig. 2C) was classified according to ginger type, with PH ginger, a large ginger bulb variety, having higher diversity than HJ ginger, a small ginger bulb variety, with the two types of ginger sharing 13,784 OTUs.

### Rank abundance curve.

**FIG 3:**
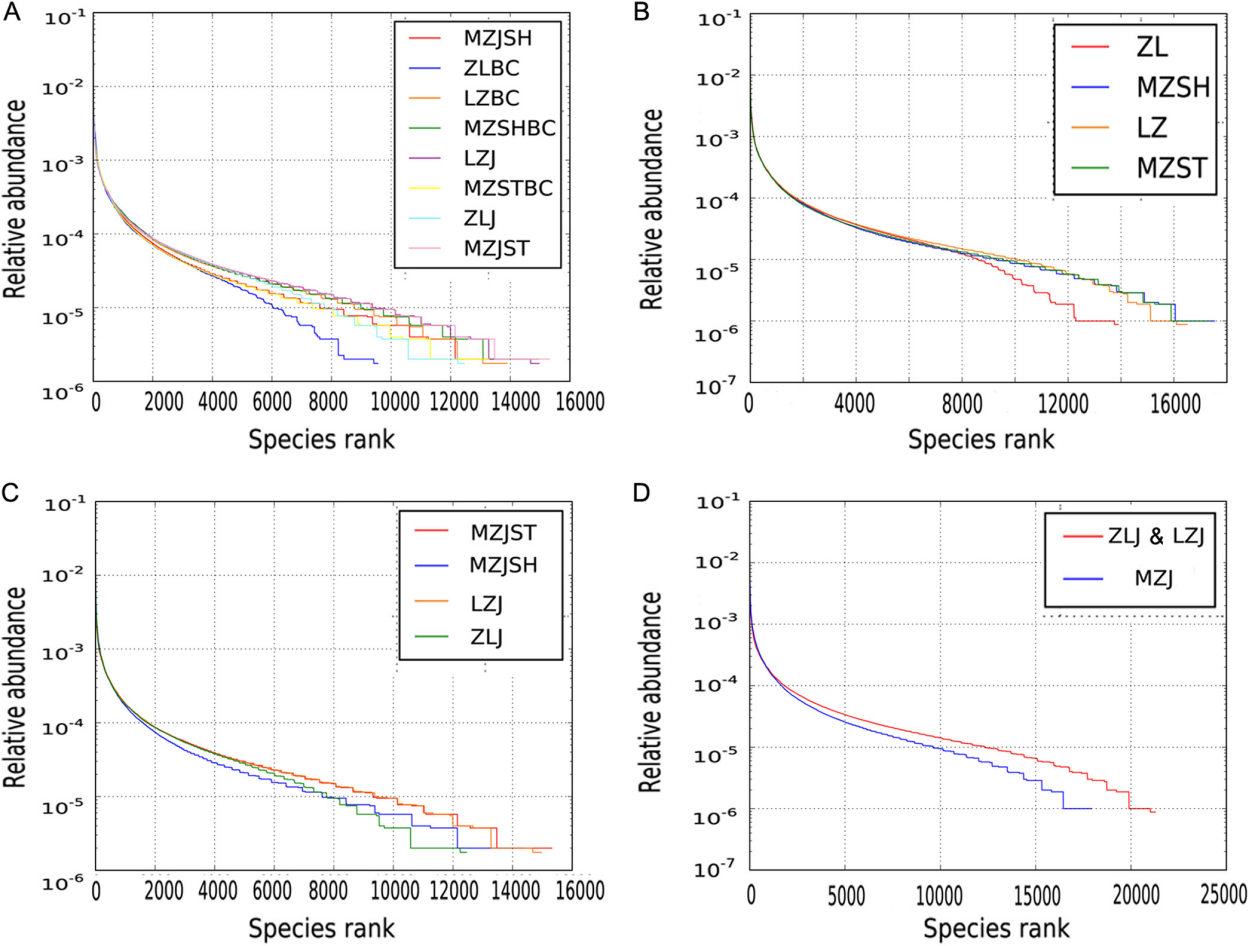
Abundance grade analyses of the OTUs of the bacterial communities in ginger soil samples. The abundance grade curve arranges the OTU in each sample according to its abundance along the abscissa and connects them (the species of OTU with the same abundance) with a broken line according to its abundance as the ordinate to reflect the distribution of OTU abundance in each sample. For each microbial community sample, the curve can directly reflect the numbers of high-abundance and rare OTUs in the community. Both the richness and evenness of the species in the sample are explained. The longer or gentler the curve, the more abundant and uniform the diversity of soil bacterial colonies in the region. The shorter or steeper the curve, the less diverse and uneven the bacterial colonies in the soil. (A) The first categorization was based on ginger and control groups. (B) The second was based on regions. (C) The third was based on the four ginger groups. (D) The fourth was based on ginger varieties (PH and HJ).

To further investigate the richness and uniformity of the bacterial species contained in each sample, we sorted the OTUs of each sample by abundance and then analyzed their abundance ranking levels. The OTU with the highest proportion in each group had a similar abundance range (from 0.1% to 1%) (Fig. 3). In contrast, the OTUs with low abundance were considerably different (Fig. 3; the length of a broken line on the horizontal axis represents the species number of OTU with this abundance).

According to category 1 (Fig. 3A), the species richness and uniformity of most groups of blank controls were less than their own corresponding ginger soil groups, indicating that the growth of ginger plants also promoted the prosperity of bacterial colony diversity. The results were contrasting for the MZSH group, probably because the MZSHBC group’s sampling site was in the vicinity of the MZJSH group, resulting in little difference between them.

In addition, according to categories 1 to 3 (Fig. 3A to C), among the ginger soil groups, MZJST had the most diverse and relatively uniform OTUs, and the ZLJ group had the least, implying that the Zhangliang region has a lower bacterial diversity than others. Regarding ginger varieties (category 4, Fig. 3D), the addition of LZJ decreased the OTU difference (diversity and uniformity) between the HJ and PH groups.

### Composition analyses of microbial communities.

**FIG 4:**
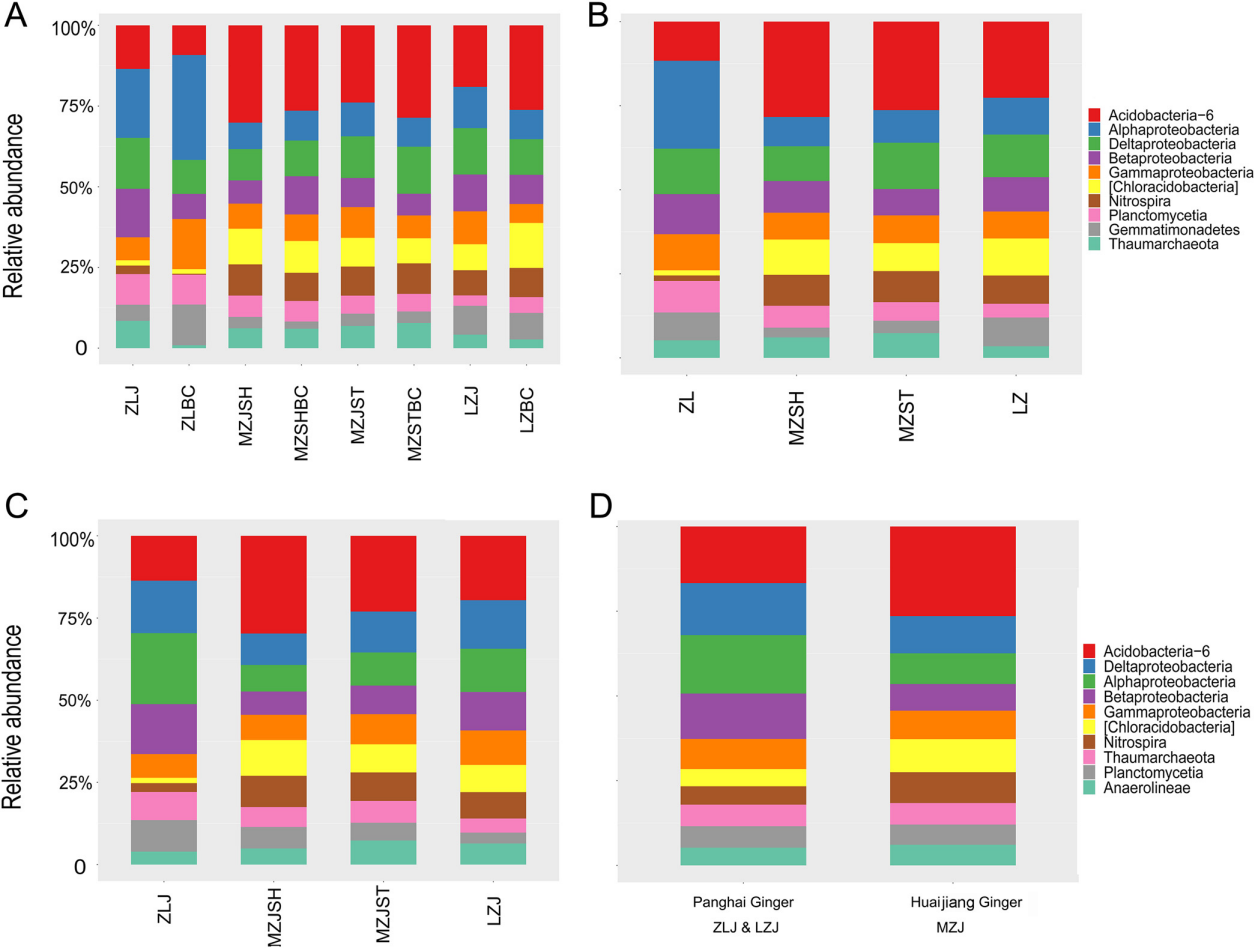
The bacterial community composition at the phylum level. The horizontal coordinate refers to each group of ginger soil bacteria. The vertical coordinate refers to the relative abundance of each group. (A) The first categorization was based on ginger and control groups. (B) The second was based on regions. (C) The third was based on the four ginger groups. (D) The fourth was based on ginger varieties (PH and HJ). It is worth noting that *[Chlorobacteria]* are a class of *Acidobacteria* phylum with a relatively large abundance.

We analyzed the dominant bacterial communities at two representative taxonomic levels for the further comparison between groups. The statistical results at both the phylum and genus levels are shown in Fig. 4 and in Fig. S2 in the supplemental material. First, at the phylum level, the top 10 dominant microorganisms (*Acidobacteria*-6, *Alphaproteobacteria*, Deltaproteobacteria, *Betaproteobacteria*, *Gammaproteobacteria*, *[Chloracidobacteria]*, *Nitrospira*, *Planctomycetia*, *Thaumarchaeota*, and *Gemmatimonadetes* or *Anaerolineae*) were similar regardless of their sampling location, ginger growth, and categorization method. However, some differences in abundance existed among bacterial categories between groups (Fig. 4). Among them, *[Chlorobacteria]* are a special class of *Acidobacteria* phylum.

According to categories 1 to 3 (Fig. 4A to C), the bacterial colony composition of the Zhangliang region (ZLJ and ZLBC) was considerably different from that of other groups. For instance, compared with other groups (Zhangliang versus others), the *Acidobacteria*-6 (~12.5% versus ~25%), *Chloracidobacteria* (~2% versus ~10%), and *Nitrospirae* (~2% versus ~10%) were less abundant in the Zhangliang region. *Acidobacteria*-6 and *Chloracidobacteria* can help ginger plants adapt to acidic ecological environments and absorb their nutrients better. For example, chemoautotrophic bacteria can take CO_2_ as a carbon source, promoting the circulation and metabolism of iron and elemental sulfur (12). *Nitrospirae* are Gram-negative bacteria; among them, nitrifiers can oxidize nitrite into nitrate, promoting nitrogen fixation, which is indispensable (12).

In addition, compared with other regions, *Alphaproteobacteria* were more abundant in the Zhangliang region (~25% versus ~11%). However, *Proteobacteria* are considered eutrophic, and *Acidobacteria* are oligotrophic (16–18), suggesting that flooding may have brought in more nutrients and promoted the propagation of eutrophic bacteria in the Zhangliang region. This may be the first consequence of the flooding and soaking. The *Gemmatimonadetes* in the ZLBC group were more abundant than in other groups. The abundance of *Thaumarchaeota* was contrasting between the groups. *Thaumarchaeota* are the only known archaea in nature besides methanogens that considerably participate in the carbon and nitrogen element cycle (12). *Thaumarchaeota* in the ZLBC group were significantly lower. For the Zhangliang region, the difference between the HJ group and the PH group became smaller due to the addition of the Liuzhai region (Fig. 4D). At the class level, the group’s classification was similar to that at the phylum level (data not shown).

At the genus level (Fig. S2), we compared the bacterial community composition of each group, especially the top nine dominant bacterial species (“*Candidatus* Nitrososphaera,” *Kaistobacter*, *Nitrospira*, *Planctomyces*, *Bacillus*, *Rhodoplanes*, *Steroidobacter*, *Gemmata*, and *Pirellula*). According to the four categorization methods, the composition of bacterial colonies in the Zhangliang region (ZLJ and ZLBC) differed significantly from that in other groups; for example, “*Candidatus* Nitrososphaera” (e.g., *Nitrospira*) is beneficial for the nitrogen fixation of ginger, but it is less abundant than others in the Zhangliang region. Meanwhile, *Kaistobacter* is more abundant than in other regions, indicating that the soil in the Zhangliang region was probably polluted by polycyclic aromatic hydrocarbons (PAHs) or heavy metals (12, 19). In addition, compared with other regions, the amount of *Geobacter* in the Zhangliang region was considerably higher than that in the other groups, indicating that the soil was probably polluted by a heavy metal (arsenic [As]) because Geobacter sulfurreducens is positively associated with the migration and transformation of arsenic in soil (20, 21). This may be another consequence of flooding and soaking.

### Genetic evolution analyses of microbial communities.

**FIG 5:**
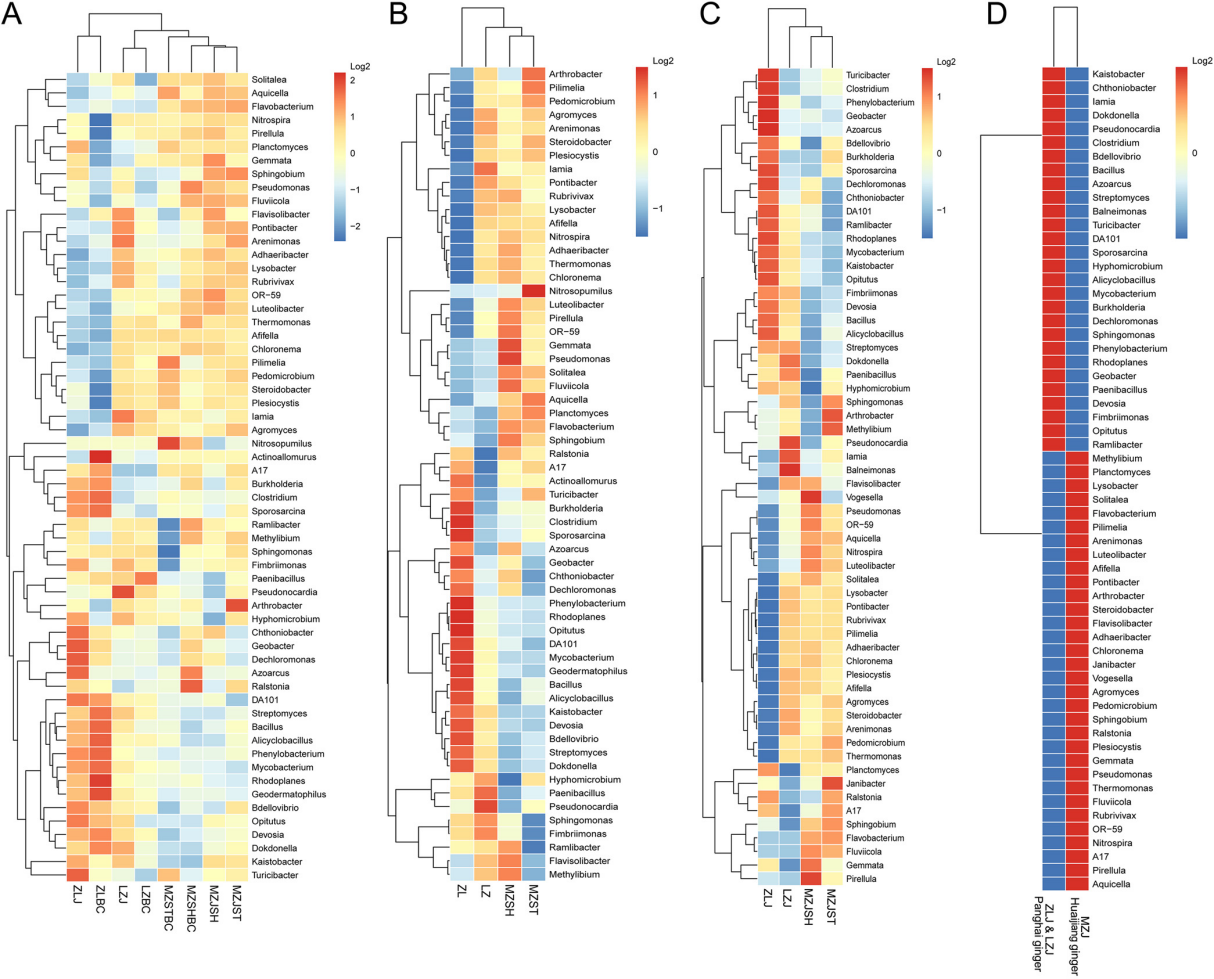
The genetic evolution analyses of microbial communities by heatmap. (Left) Genetic evolutionary tree lines. (Right) The top 60 microbial communities (at the genus level) in abundance. (Top) The clustering relationships between the samples. (Bottom) Sample names. (A) The first categorization was based on ginger and control groups. (B) The second was based on regions. (C) The third was based on the four ginger groups. (D) The fourth was based on ginger varieties (PH and HJ).

To display the genetic evolution relationship of bacterial communities in each group, we conducted genetic evolution analyses of the top 60 microbial communities in abundance at the genus level using heat maps (Fig. 5). The branch lines of genetic evolution at the top of the figure indicate that the soil bacterial communities from the same region can always cluster together, implying that the closer the sampling location is, the more similar the soil bacterial communities are (Fig. 5A). Especially for category 1, although MZSTBC’s and MZSHBC’s soil types were inconsistent, they were clustered together because they belonged to the same Mengzhou region. For category 2 (Fig. 5B), the differences in the bacterial communities between regions were more significant, with almost all bacterial species having different abundances in the four sampling sites. The Zhangliang and Liuzhai regions showed almost opposite microbial abundances, although their ginger species were the same. Category 3 results (Fig. 5C) showed a similar phenomenon. The results of category 4 indicated that the bacterial colony structures of the soil in Mengzhou (HJ ginger) and non-Mengzhou (PH ginger) regions are very different (Fig. 5D). The genetic evolutionary tree line on the left represents the species of bacteria (with names on the right) clustered together or classified in several regions.

### Diversity analyses.

**FIG 6:**
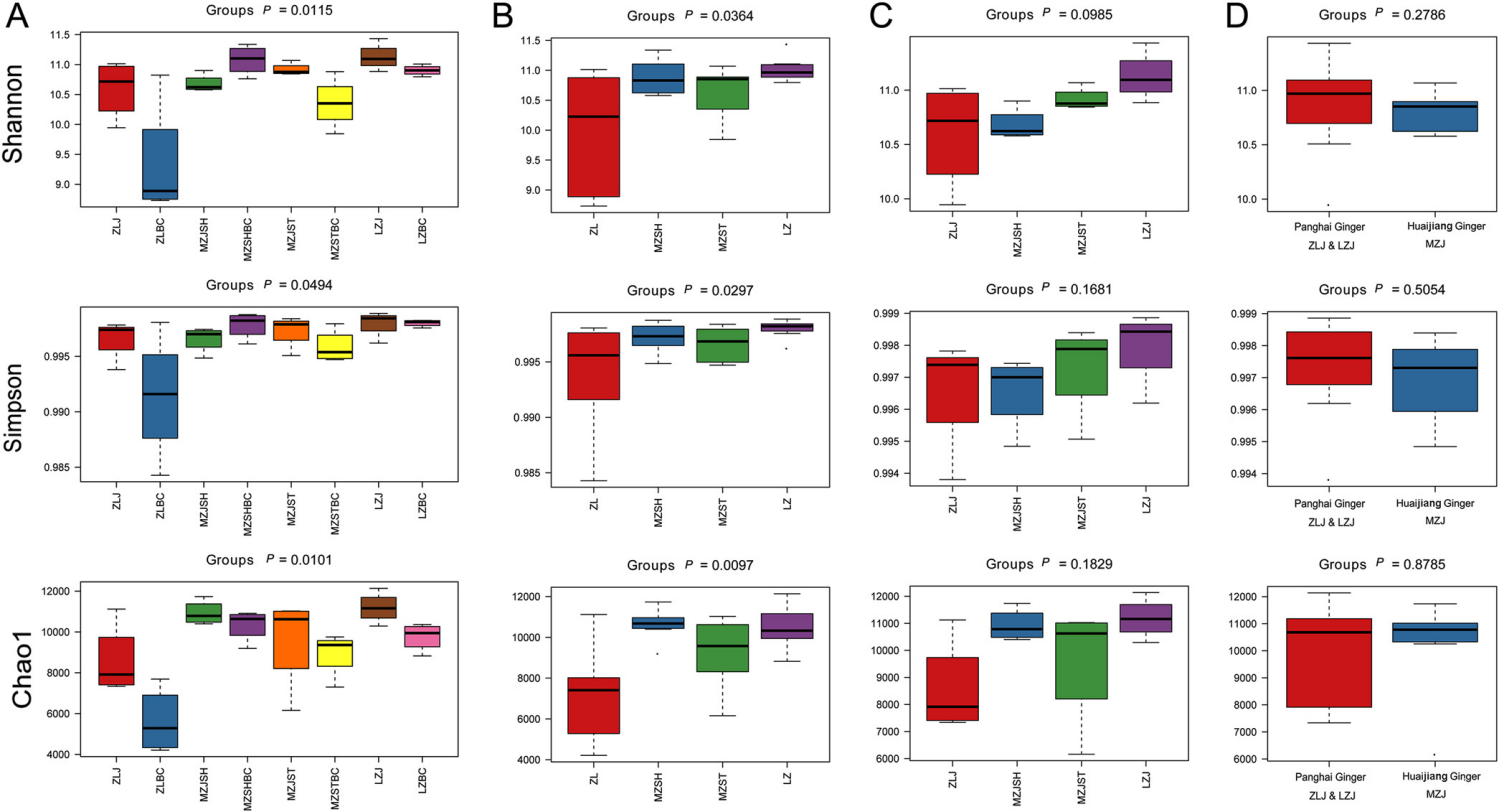
α-Diversity analyses by box plots. Three indexes were used to evaluate α-diversity: Shannon entropy, Simpson index, and Chao index (from top to bottom). (A) The first categorization was based on ginger and control groups. (B) The second was based on regions. (C) The third was based on the four ginger groups. (D) The fourth was based on ginger varieties (PH and HJ). Comparisons between groups were performed using the Kruskal test in R. A *P* value of ≤0.05 was considered statistically significant.

To assess the richness and diversity of the soil bacterial community of ginger, we first calculated the values of α-diversity of the samples using the Quantitative Insights into Microbial Ecology (QIIME, v1.9.0) software and three indexes and evaluated the significant differences between groups (Fig. 6). In the cases of category 1 and category 2 (Fig. 6A and B), the three indexes showed significant differences in diversity between groups (*P* < 0.05). The other two categorization methods (Fig. 6C and D) showed no significant difference in the α-diversity (*P* > 0.05). Besides MZSHBC, the diversities in other blank controls were all slightly lower than that in ginger groups (although there might be no significant difference), which was consistent with the above results, also indicating that the growth of ginger plants promoted bacterial diversity. In contrast, between the ginger groups (Fig. 6C), the LZJ group had the highest α-diversity (although the *P* value was greater than 0.05), which could be attributed to the ginger and cherry intercropping method (Table 2), which usually promotes improved soil quality and microbial diversity (14, 22).

**TABLE 2:** Sampling information^*a*^

Soil no.	ID	Ginger species	Ginger ball size	Tillage method	Soil type	Location	Sampling date (yr.mo.day)
1	ZLJ01-04	Panghai ginger from Changyi city	Large	Crop rotation; monoculture	Sandy soil	Zhangliang (Lushan city); longitude, 113.07; latitude, 33.69	2021.09.30
2	ZLBC01-04	Panghai ginger from Changyi city	Large	NA	Sandy soil	Zhangliang (Lushan city); longitude, 113.07; latitude, 33.69	2021.09.30
3	LZJ01-04	Panghai ginger from Changyi city	Large	Interplanting with cherry	Red clay soil	Liuzhai (Xinmi city); longitude, 113.57; latitude, 34.52	2021.10.06
4	LZBC01-04	Panghai ginger from Changyi city	Large	NA	Red clay soil	Liuzhai (Xinmi city); longitude, 113.57; latitude, 34.52	2021.10.06
5	MZJST01-04	Huaijiang ginger from Jiaozuo city	Small	Monoculture	Sandy soil	Gudan (Mengzhou city); longitude, 112.80; latitude, 34.98	2021.10.07
6	MZSTBC01-04	Huaijiang ginger from Jiaozuo city	Small	NA	Sandy soil	Gudan (Mengzhou city); longitude, 112.80; latitude, 34.98	2021.10.07
7	MZJSH01-04	Huaijiang ginger from Jiaozuo city	Small	Crop rotation; monoculture	Mixed soil of sandy and dark loessal soils	Gudan (Mengzhou city); longitude, 112.80; latitude, 34.98	2021.10.07
8	MZSHBC01-04	Huaijiang ginger from Jiaozuo city	Small	NA	Mixed soil of sandy and dark loessal soils	Gudan (Mengzhou city); longitude, 112.80; latitude, 34.98	2021.10.07

a We collected two types of ginger, PH and HJ, and they were planted in three types of soils: sandy, red clay, and combination (dark loessal and sandy soils) in three cities of Henan Province, China. Four ginger soil groups were set up with their blank controls (BC), each group containing four samples, totaling 32 samples. They were named ZLJ01-04, ZLBC01-04, MZJST01-04, MZSTBC01-04, MZJSH01-04, MZSHBC01-04, LZJ01-04, and LZBC01-04. The experimental group was sampled in the ginger planting region; the blank group was sampled in the adjacent nonplanting region. The nomenclature rules were as follows: ZL, MZ, and LZ refer to the sampling sites Lushan (Zhangliang township), Mengzhou (Gudan township), and Xinmi (Liuzhai township), respectively. J refers to the Chinese “jiang” (ginger) abbreviation. ST refers to sandy soil; SH refers to the combination of sandy and dark loessal soil. ID, identifier; NA, not applicable.

**FIG 7:**
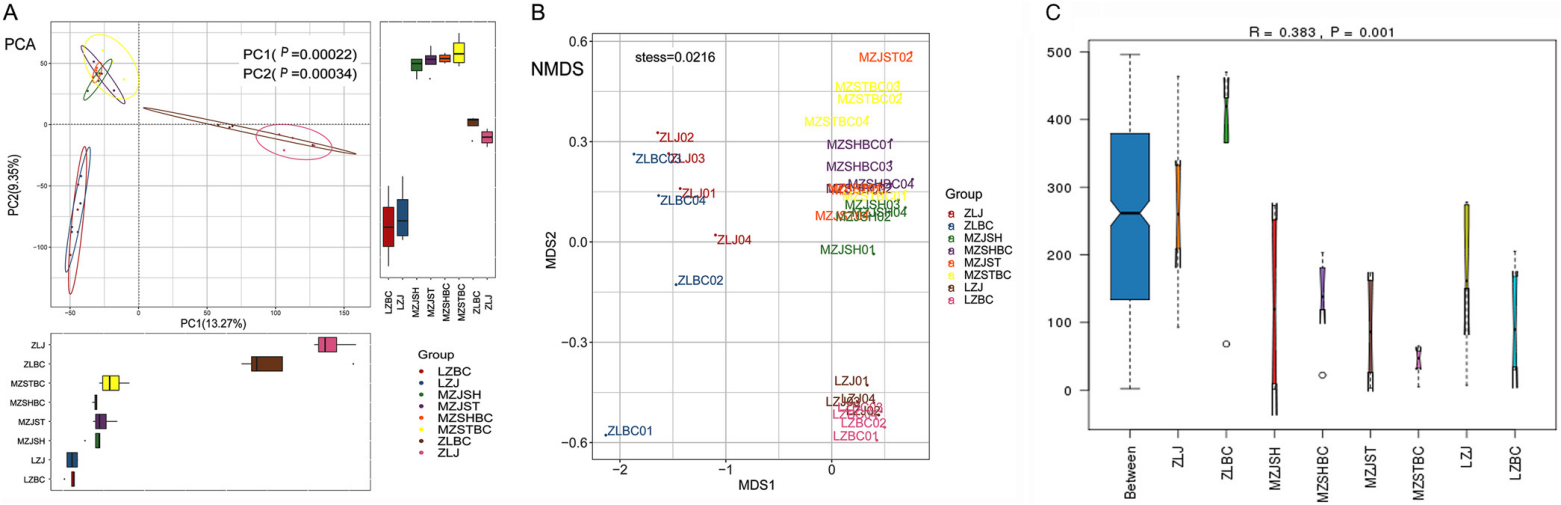
β-Diversity and similarity analyses. Two statistical methods, PCA and NMDS, were performed to compare the β-diversity between multiple groups. (A) PCA; the *P* values between the groups were shown in both PC1 (horizontal axis) and PC2 (vertical coordinate). (B) NMDS; the above analysis results showed clustering and separation effects between groups. (C) ANOSIM; the similarity analyses in the microbial communities between groups were performed. Comparisons between groups were performed using the Kruskal test in R. A *P* value of ≤0.05 was considered statistically significant. The naming rules are the same as described above.

To further compare the β-diversities between multiple groups, i.e., to examine the categorization method effects using OTU data, we performed two statistical methods, PCA and nonmetric multidimensional scaling (NMDS) (Fig. 7). The results of category 1 are used as examples here. As shown in the PCA plot (Fig. 7A), the aggregation effect of samples from the same region was evident. The clustering ellipses of the regional samples did not exhibit any overlap. For instance, although MZST and MZSH samples were collected from different sites (2 to 3 km apart), they were clustered together (Fig. 7A, upper left) because they belonged to the same region (Gudan township of Mengzhou city). The samples from the Zhangliang region clustered together at the right of Fig. 7A and those of the Liuzhai region at the bottom left. The *P* values between the groups were significant (*P* < 0.001) in both PC1 and PC2 dimensions. In addition, the separation effect between the groups was satisfactory.

In addition, NMDS analyses results showed similar clustering and separation effects (Fig. 7B). The similarity in the microbial communities within one group (from the same region) was high, and the differences between the groups (from different regions) were also obvious. Also, the analyses of similarities (ANOSIM) (Fig. 7C, *R* = 0.383 > 0 and *P* = 0.001) suggested that the differences between the groups were greater than the differences within the groups. Besides, the ANOSIM results also indicate that the categorization method of the samples based on region is meaningful and appropriate.

### Species marker screening.

**FIG 8:**
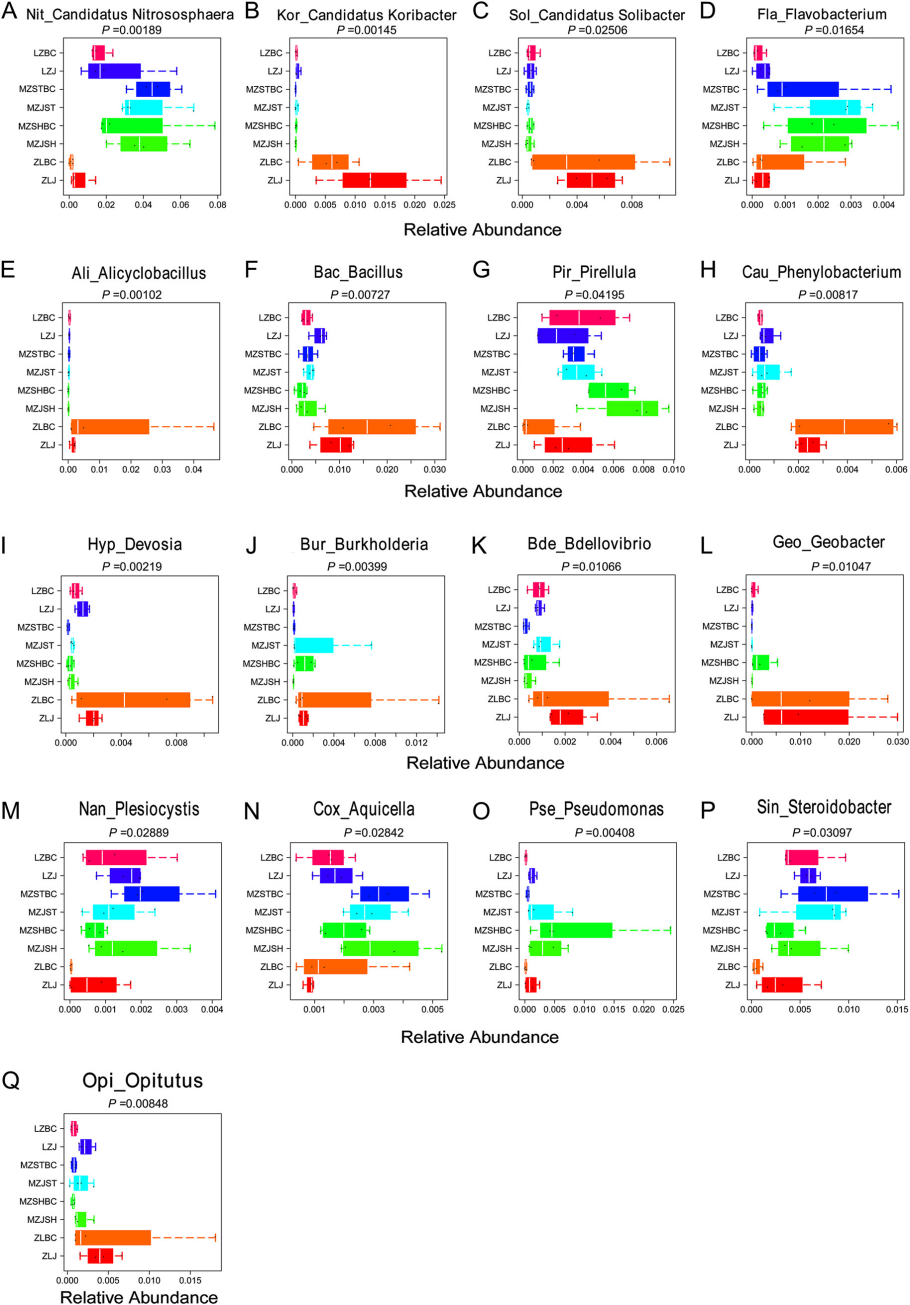
Species marker analyses at the genus level. The different colors in the diagram represent different groupings. Each box plot represents a species, and the species name is shown at the top of the plot. The relative abundance is shown on the horizontal coordinate. The vertical coordinate represents the sample names. Only the results of the analyses of the species-specific markers of the first category (category 1) are shown as an example.

To screen species markers between groups for each subgroup, we performed an analysis of species differences at the genus level, i.e., species with significant differences between subgroups were selected as markers. Box plots in Fig. 8 show that some species with significant differences (using Kruskal test in R) between groups were screened, including “*Candidatus* Nitrososphaera” (which can be used as a species marker in non-Zhangliang regions) (Fig. 8A, *P* = 0.00189). *Alicyclobacillus* (Fig. 8E, *P* = 0.00102), *Bacillus* (Fig. 8F, *P* = 0.00727), *Phenylobacterium* (Fig. 8H, *P* = 0.00817), *Devosia* (Fig. 8I, *P* = 0.00219), *Burkholderia* (Fig. 8J, *P* = 0.00399), *Bdellovibrio* (Fig. 8K, *P* = 0.01066), and Opitutus (Fig. 8Q, *P* = 0.00848) can be used as markers of the ZLBC group (Fig. 8E, *P* = 0.00102). *Flavobacterium* can be used as a marker of the Mengzhou region (Fig. 8D, *P* = 0.01654). “*Candidatus* Koribacter” (Fig. 8B, *P* = 0.00145), “*Candidatus* Solibacter” (Fig. 8C, *P* = 0.02506), and *Geobacter* (Fig. 8L, *P* = 0.01047) can be used as regional markers of the Zhangliang region. In addition, the relative abundances of *Plesiocystis* (Fig. 8M, *P* = 0.02889), *Aquicella* (Fig. 8N, *P* = 0.02842), and Pseudomonas (Fig. 8O, *P* = 0.00408) also differed significantly between the groups. No obvious markers were found for the soil bacterial community in the Liuzhai region.

Subsequently, we used a selection of species markers using the linear discriminant analysis effect size (LEfSe) method (based on linear discriminant analysis) and the GraPhlan method (sample community distribution map of species evolutionary tree [data not shown]). The LEfSe analyses combine linear discriminant analyses with nonparametric Kruskal-Wallis and Wilcoxon rank sum tests to screen for biomarkers (species that differ significantly between groups) between groups. The results of this analysis of dominant species (interspecific markers) were slightly different from those of box line plots, but there was some correlation.

Consistent with the above results, *Alicyclobacillus*, *Bacillus*, *Phenylobacterium*, *Devosia*, *Burkholderia*, and *Bdellovibrio* were also classified as ZLBC markers (see Fig. S3 in the supplemental material). Compared to the box plot (Fig. 8D), *Flavobacterium* was further classified as a marker of the MZJST group (Fig. S3). “*Candidatus* Nitrososphaera” was further classified as a marker of the MZJSH group. Furthermore, the LEfSe analyses present the respective intergroup markers for the ZLJ and LZJ groups (not given in the box plots above). *Elusimicrobia* was classified as a marker of the ZLJ group, and *Actinomycetospora* as a marker of the LZJ group (Fig. S3).

### Prediction of the metabolic function of bacteria.

To predict the metabolic functions of the bacterial communities, based on BugBase software, we performed a comparison of phenotypic classifications between groups, including Gram positivity, Gram negativity, biofilm formation, pathogenicity, mobile components, oxygen demand (including anaerobic, aerobic, and parthenogenic bacteria), and oxidative stress tolerance (seven categories), taking only the first category method as the example (Fig. S4).

As shown, there existed significant differences between groups of aerobic (Fig. S4A), Gram-negative (Fig. S4F), Gram-positive (Fig. S4G), and stress-tolerant (Fig. S4I) bacteria (*P* < 0.01). In particular, the relative abundance of the bacterium in the ZLJ and ZLBC groups showed significant differences from others. This result indicates that after flooding and soaking, the soil bacteria in the Zhangliang region suffered from a more complex soil environment than other groups, requiring more aerobic and stress-tolerant bacteria to maintain the metabolic cycle of vital elements and the nutritional requirements of the ginger plants. This is consistent with the Zhangliang region suffering from more severe flooding than other regions.

## DISCUSSION

The bacterial diversity of the soil was restored with timely drainage 2 months after the flooding and inundation on 20 July 2021 (15). In this study, we used 16S rRNA high-throughput sequencing and bioinformatic analyses to compare the bacterial community structures of characteristic ginger soils from three regions in the Henan Province of north-central China. The species of the top 10 bacterial phyla showed high similarity with those previously reported (13), but each accounted for different percentages. With probably the most severe flooding, the Zhangliang region’s bacterial community structures differed significantly from others. The bacterial diversity in Zhangliang was relatively lower than others, although the flooding promoted the propagation of eutrophic bacteria. The lands of the Zhangliang region may also be contaminated by PAHs or heavy metals, indicating that the effect of flood soaking on soil microorganisms is twofold. Importantly, the sampled lands of the Zhangliang region require improved management in the future.

PCA showed that the sampling location had the greatest influence on the soil bacterial community characteristics, and the soil bacterial community structure was most similar when sampled from the same region. In this study, although the soil types of MZST (sandy soils) and MZSH (combination) regions were different, their PCA clustered them together. Meanwhile, although the ginger types of ZLJ and LZJ were the same, their PCA results were distinguishing. This result shows that the structure of the soil bacterial community is mainly determined by the geographical location of the sample (23) and its associated climate, environment, and farming practices, and not mainly by the ginger variety. In other words, geographical location influenced the microbial populations more than did soil type or ginger variety, consistent with a previous study (23).

Although the Zhangliang region had the lowest bacterial diversity, the number of unique OTUs was the highest. According to the investigation, the ZLJ group had a large planting region (~1 km^2^) and inadequate drainage and was most severely affected by flooding; there may be a compensatory increase in aerobic and stress-tolerant bacteria. This suggests that some OTUs may not be ginger soil-specific microorganisms in the region but are present due to the flooding. In contrast, Mengzhou had a smaller cultivation region (0.005 km^2^), drained more rapidly, and was less affected; hence, the unique OTUs in the region may be the microorganisms specific to this region.

The unique OTU analyses may also help enhance the brand characteristics of ancient HJ ginger, thereby improving its brand value. In conclusion, these findings help us understand the species and composition of bacteria (3, 7) and infection of ginger soil after flooding and soaking. They also guide the significance of conserving ancient characteristic ginger varieties and enhancing biological control of plant bacterial diseases (3, 13).

## MATERIALS AND METHODS

### Sample information.

Two ginger species, PH and HJ, were planted in three types of soil: sandy, red clay, and combination (dark loessal and sandy soils) in three cities of Henan province, China. In total, four ginger (J, short for “jiang” in Chinese) groups were set up with their corresponding blank controls (blank soil in nearby field paths with no crops), each group with four samples, totaling 32 samples (Table 2). They were named ZLJ01-04, ZLBC01-04, MZJST01-04, MZSTBC01-04, MZJSH01-04, MZSHBC01-04, LZJ01-04, and LZBC01-04. The experimental group was sampled in the ginger planting region; the blank group was sampled in the adjacent nonplanting region (Table 2). When sampling, we first removed the surface floating soil and dug the subsurface 15-cm layer of soil using an ethanol-sterilized fire shovel. After removing visible impurities, each sample group was collected from the four sites, and an appropriate amount of soil samples was loaded in sterile 2.0-mL centrifuge tubes, each tube accommodating approximately 300 mg. Then, they were immediately kept at −80°C or on dry ice for cryopreservation and transportation.

### Soil bacterial gDNA extraction and 16S rRNA pyrosequencing.

Total genomic DNA (gDNA) of bacterial soil samples was extracted using the gDNA isolation kit (TianGen, Beijing, China) according to the manufacturer’s instructions. The quality and quantity of extracted gDNA were determined using agarose gel electrophoresis and Nanodrop 2000 (Thermo Fisher Scientific, Waltham, MA, USA), respectively. The gDNA was stored at −20°C before further assessment.

The PCR amplification of the bacterial V4 regions of the 16S rRNA genes was performed using the upstream primer 5′-GTGCCAGCMGCCGCGGTAA-3′ and the downstream primer 5′-GGACTACHVGGGTWTCTAAT-3′. In order to differentiate the sample, the specific paired-end (PE) barcode (6 bp) was fused into the TruSeq adaptor for high-throughput sequencing. The amplification reaction system (total, 50 μL) contained 25 μL Phusion high-fidelity master mix, 3 μL (10 μM) each primer, 10 μL gDNA (as the template), 3 μL dimethyl sulfoxide, and 6 μL double-distilled water (ddH_2_O). The PCR cycle comprised initial denaturation (98°C for 30 s), followed by 26 cycles of denaturation (98°C for 15 s), annealing (58°C for 15 s), and extension (72°C for 15 s), with a final extension (72°C for 60 s). The ~450-bp PCR products were purified using AMPure XP beads (Beckman Coulter, Indianapolis, IN, USA) and quantified by the Qubit dsDNA HS assay kit (Life Technologies, Gaithersburg, MD, USA). After quantification, all amplicons were pooled in equal amounts (50 ng), and PE 150-bp sequencing was performed using the NovaSeq6000 (Illumina, San Diego, CA, USA) at GUHE Info Technology Co., Ltd. (Hangzhou, China).

### Data analyses.

We utilized QIIME software to process the sequencing reads, as previously reported (24). In brief, the raw reads that matched the barcodes were assigned to each soil sample and further identified as valid data. Besides, low-quality reads with average Phred scores of <20, <151-bp lengths, ambiguous bases, and mononucleotide repeats of >8 bp were filtered out (25, 26). The PE reads were merged by overlap, and OTUs were picked using Vsearch v2.4.4 (--fastq_mergepairs --fastq_minovlen 5) (27), which included cluster (--cluster_fast, --id 0.97), dereplication (--derep_fulllength), and detection of chimeras (--uchime_ref). Next, we selected a representative sequence from each OTU using the default parameters and used Vsearch to search the Greengenes database for representative sequences for OTU classification.

A table recording the taxonomy and abundance of each OTU for each sample was created. OTUs with an <0.001% abundance of total sequences across all samples were discarded. To minimize the variation in sequencing depth across samples, an averaged, rounded rarefied OTU table was also generated by averaging a subset of 100 uniformly resampled OTUs below 90% of the minimum sequencing depth for further analyses.

### Bioinformatics.

The bioinformatics analyses of the sequencing reads were mainly performed using R packages (v3.2.0) and QIIME. α-Diversity indexes based on OTU level, including the Shannon diversity index, Chao1 richness estimator, and Simpson index, were calculated using the OTU table in QIIME. The ranked abundance curves based on OTU level were generated to compare the richness and evenness of OTUs between groups.

The structural variations of microbial communities between groups were also investigated by β-diversity analyses based on OTU level, using UniFrac distance metrics (28, 29) and nonmetric multidimensional scaling (NMDS) (30). PCA was also performed based on the genus-level compositional profiles (30). The significance of structural differentiation of bacterial communities between groups was evaluated by permutational multivariate analysis of variance (PERMANOVA) using the R package “Vegan” (31). Venn diagrams were generated using the R package “Venn Diagram” to visualize shared and unique OTUs between groups, based on the occurrence of OTUs between groups, regardless of their relative abundance (32).

Taxon abundances (on the phylum and genus levels) were statistically compared between groups using the Kruskal test in the R statistical package. Linear discriminant analysis effect sizes (LEfSes) were performed to detect taxa with different abundances between groups using default parameters (33). Then, we applied random forest analyses to distinguish the different samples and groups using the R package “randomForest” with 1,000 trees, with all the settings at the default (34). The generalization error was estimated using the 10-fold cross-validation method. The expected “baseline” error was also included, obtained from the classifier that predicts the most common category label. Cooccurrence analyses were performed by calculating Spearman’s rank correlations between predominant taxa. Meanwhile, correlations with |RHO| of >0.6 and a *P* value of <0.01 were visualized as cooccurrence networks using Cytoscape (35). We had predicted microbial functions by phylogenetic investigation of communities by reconstruction of unobserved states (PICRUSt) based on high-quality reads (36). Besides, we had used the statistical analysis of metagenomic profiles (STAMP) software package v2.1.3 for further analyses of the output files (37). BugBase is an excellent software program for the measurement of high-level phenotypes within the microbiome (38). FAPROTAX is a data bank that maps prokaryotic clades (e.g., genera or species) to establish metabolic or other ecologically relevant functions (39).

### Ethics approval and consent to participate.

This study did not involve human or animal experimental samples. We confirm that all experiments were conducted in accordance with relevant guidelines and regulations.

### Data availability.

The supporting data have been deposited into the Sequence Archive of China National GenBank database (40, 41) with the accession number CNP0003539.
