# Supplementary material for: Comparative Analyses of Soil Bacterial Colonies of Two Types of Chinese Ginger after a Major Flood Disaster
Source: Microbiol Spectr. 2023 Feb 6;11(2):e04355-22. doi: 10.1128/spectrum.04355-22 (PMC10100910; doi:10.1128/spectrum.04355-22)
Supplement: Supplementary file 1 — Fig. S1 to S4 and list of abbreviations. Download spectrum.04355-22-s0001.pdf, PDF file, 14.8 MB [file spectrum.04355-22-s0001.pdf]

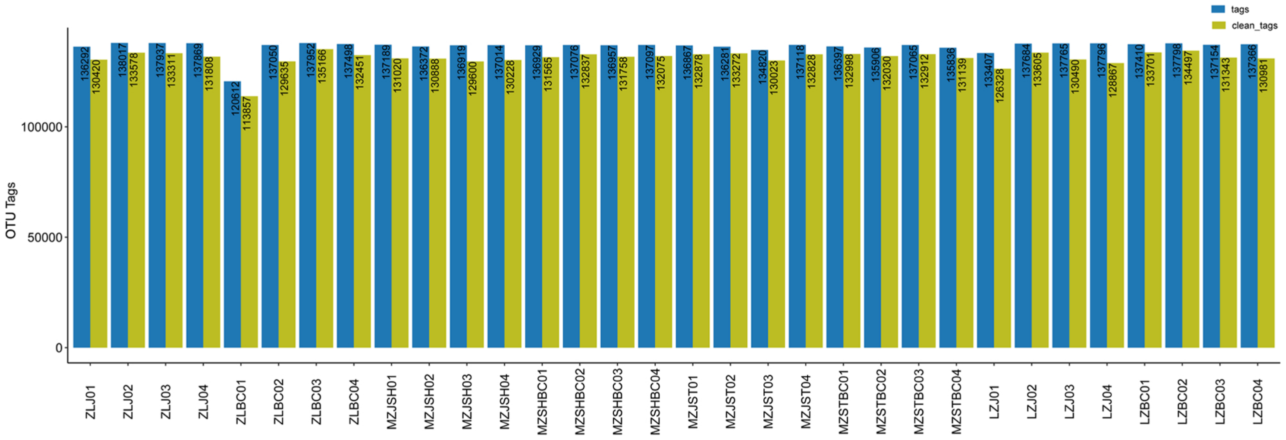
tags
clean_tags
OTU Tags
0
50000
100000
136292
130420
138017
133578
137937
133311
137869
131808
120612
113857
137050
129635
137952
135166
137498
132451
137189
131020
136372
130888
136919
129600
137014
130228
136929
131565
137076
132837
136957
131758
137097
132075
136867
132878
136281
133272
134820
130023
137118
132828
136397
132998
135906
132030
137065
132912
135836
131139
133407
126328
137684
133605
137765
130490
137796
128867
137410
133701
137798
134497
137154
131343
137366
130981
ZLJ01
ZLJ02
ZLJ03
ZLJ04
ZLBC01
ZLBC02
ZLBC03
ZLBC04
MZJSH01
MZJSH02
MZJSH03
MZJSH04
MZSHBC01
MZSHBC02
MZSHBC03
MZSHBC04
MZJST01
MZJST02
MZJST03
MZJST04
MZSTBC01
MZSTBC02
MZSTBC03
MZSTBC04
LZJ01
LZJ02
LZJ03
LZJ04
LZBC01
LZBC02
LZBC03
LZBC04

Figure S1 The tag statistics for each ginger soil bacterial sequencing data. The horizontal coordinate refers to each ginger soil bacteria, namely ZLJ01-04, ZLBC01-04, MZJST01-04, MZSTBC01-04, MZJSH01-04, MZSHBC01-04, LZJ01-04 and LZBC01-04. The naming rules are as follows: ZL, MZ, and LZ refer to the sampling sites: Lushan (Zhangliang), Mengzhou, and Xinmi (Liuzhai), respectively. J refers to the abbreviation of the Chinese "Jiang (ginger)." ST, sandy soil; SH, a combination of sandy and dark loessial soils; BC, blank control. The vertical coordinate refers to the tags acquired from the results of NGS data filtering and processing. Tags are the sequencing reads containing the correct overlaps after quality filtering, along with correct barcodes and high-quality V4 regions.

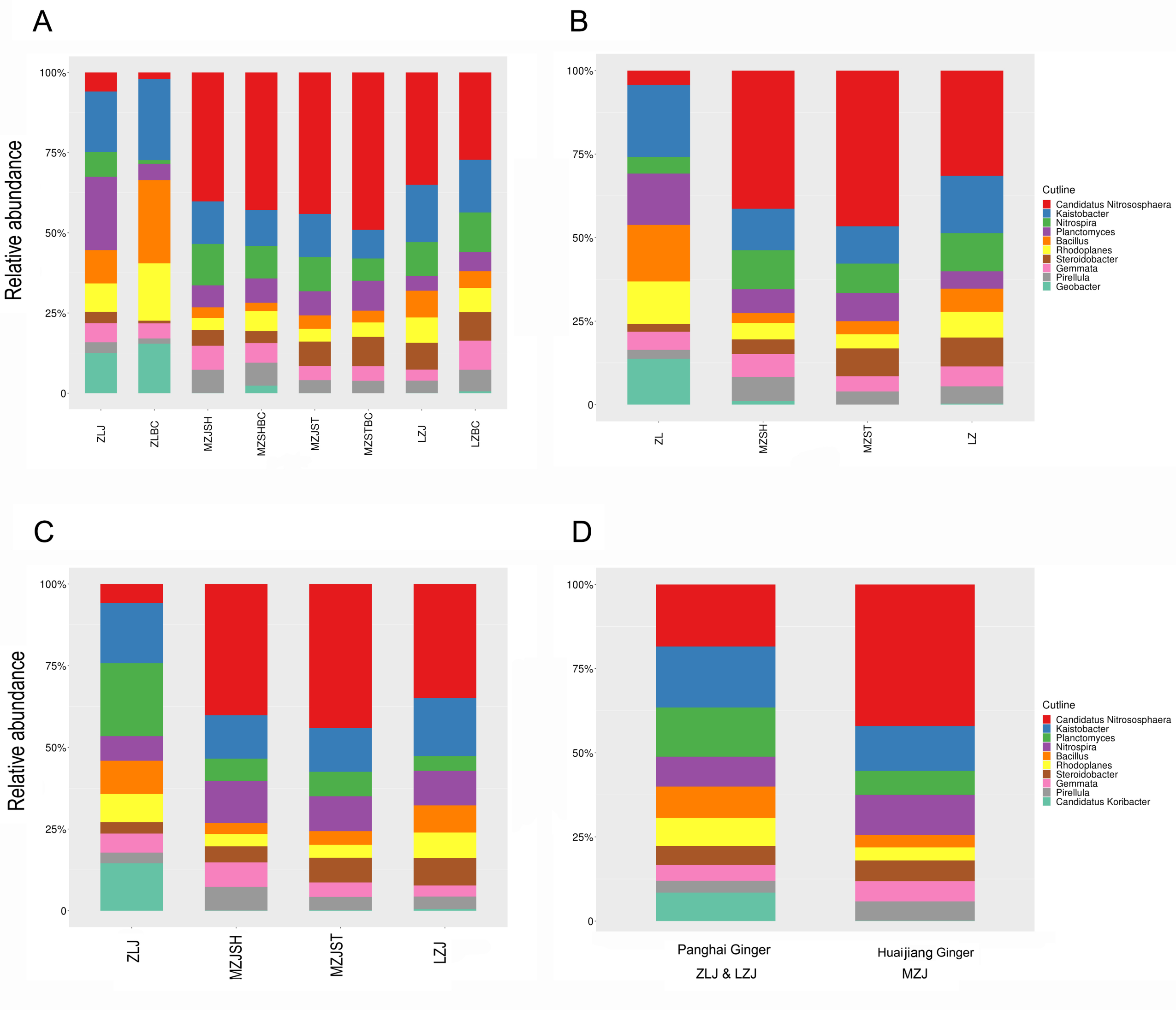
A
B
C
D
Relative abundance
100%
75%
50%
25%
0
ZLJ
ZLBC
MZJSH
MZSHBC
MZJST
MZSTBC
LZJ
LZBC
ZL
MZSH
MZST
LZ
Cutline
Candidatus Nitrososphaera
Kaistobacter
Nitrospira
Planctomyces
Bacillus
Rhodoplanes
Steroidobacter
Gemmata
Pirellula
Geobacter
Relative abundance
ZLJ
MZJSH
MZJST
LZJ
Panghai Ginger
ZLJ & LZJ
Huaijiang Ginger
MZJ
Cutline
Candidatus Nitrososphaera
Kaistobacter
Planctomyces
Nitrospira
Bacillus
Rhodoplanes
Steroidobacter
Gemmata
Pirellula
Candidatus Koribacter

Figure S2 The bacterial community composition at the genus level. The horizontal coordinate refers to each group of ginger soil bacteria. The vertical coordinate refers to the relative abundance of each group. There are four different categorization methods included. **A.** The first categorization was based on ginger & control groups. **B.** The second was based on regions. **C.** The third was based on the four ginger groups. **D.** The fourth was based on ginger varieties (PH and HJ). The naming rules are the same as described above.

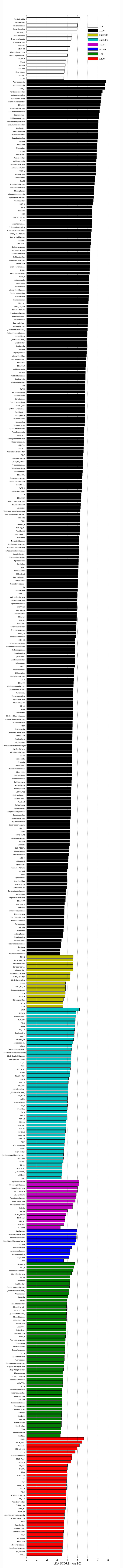

ZLJ
ZLBC
MZSTBC
MZSHBC
MZJST
MZJSH
LZJ
LZBC
0
1
2
3
4
5
6
7
8
LDA SCORE (log 10)

Figure S3 LEfSe analyses for species markers. LEfSe is based on linear discriminant analysis (LDA), which combines linear discriminant analysis with non-parametric *Kruskal–Wallis* and *Wilcoxon rank-sum tests* to screen for biomarkers (species that differ significantly between groups). The different colors in the graph represent the significantly different species between the different groups. The analysis was obtained using the LefSe software, where the logarithmic LDA score for significant differences was set to 2. The horizontal coordinate refers to the LDA score (log_{10}); the higher the score, the more appropriate the species is as a marker; the vertical coordinate is the name of the marker species in each group. The naming was the same as described above. Only the results of the analyses of the first category (Category1) are shown as examples.

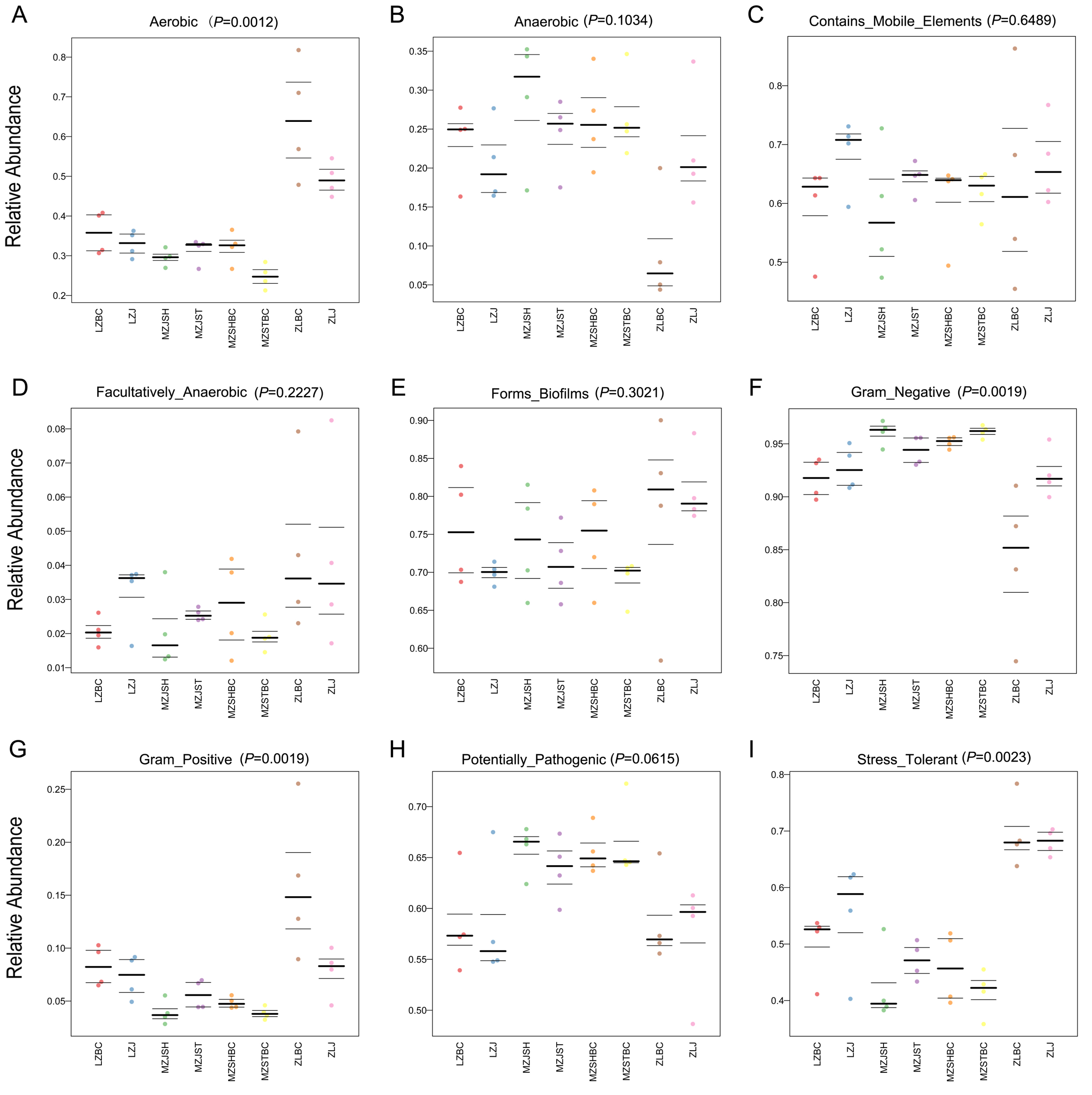

A
Aerobic (P=0.0012)
B
Anaerobic (P=0.1034)
C
Contains_Mobile_Elements (P=0.6489)
D
Facultatively_Anaerobic (P=0.2227)
E
Forms_Biofilms (P=0.3021)
F
Gram_Negative (P=0.0019)
G
Gram_Positive (P=0.0019)
H
Potentially_Pathogenic (P=0.0615)
I
Stress_Tolerant (P=0.0023)
Relative Abundance
LZBC
LZJ
MZJSH
MZJST
MZSHBC
MZSTBC
ZLBC
ZLJ

Figure S4 Prediction of metabolic function of bacteria. A comparison of phenotypic classifications between groups, including aerobic (**A**), anaerobic (**B**), containing mobile elements (**C**), facultative anaerobic (**D**), biofilm-forming (**E**), gram-negative (**F**), gram-positive (**G**), potentially parthenogenic bacteria (**H**), and oxidative stress-tolerant (**I**), only taking the first category method (Category1) as the example. The horizontal coordinate represents the sample name. The relative abundance is shown in the vertical coordinate. Comparisons between groups were performed using the *Kruskal test* in R. $P \leq 0.05$ was considered statistically significant.

List of Abbreviation

16S rRNA: It is the DNA sequence corresponding to the RNA encoding the ribosome (16S subunit) in bacteria and is present in the genome of all bacteria;

ANOSIM: The analyses of similarities;

CO2: Carbon dioxide;

GraPhlan: It is a software tool for producing high-quality circular representations of taxonomic and phylogenetic trees.

HJ: Chinese ginger variety-Huaijiang ginger;

LEfSe: Linear discriminant analysis effect sizes;

Liuzhai (LZ): It refers to the following two groups (LZJ and LZBC); Liuzhai is the name of a township in Xinmi city, Henan province, China;

LZJ: Red clay soil sampled from Liuzhai (Panghai ginger); J: It refers to the Chinese "jiang", Which is ginger in Chinese;

LZBC: Red clay soil sampled from Liuzhai (blank control);

Mengzhou (MZ): Mengzhou is the name of a city in Henan province, China; herein it is specifically referring to the Gudan township of Mengzhou;

MZJSH: Mixed soil sampled from Mengzhou (Huaijiang ginger);

MZSHBC: Mixed soil sampled from Mengzhou (blank control);

MZSH: It refers to the above two groups (MZJSH and MZSHBC);

MZJST: Sandy soil sampled from Mengzhou (Huaijiang ginger);

MZSTBC: Sandy soil sampled from Mengzhou (blank control);

MZST: It refers to the above two groups (MZJST and MZSTBC);

NGS: The next generation sequencing;

NMDS: The nonmetric multidimensional scaling analyses method;

OTU: Operational taxonomic unit;

PAH: polycyclic aromatic hydrocarbon;

PCA: Principal component analysis;

PE: Paired-end;

PH: Chinese ginger variety-Panghai ginger;

QIIME: The Quantitative Insights into Microbial Ecology software;

V4: The fourth variable region of the 16S rRNA;

ZLJ: Sandy soil sampled from Zhangliang (Panghai ginger);

ZLBC: Sandy soil sampled from Zhangliang (blank control);

Zhangliang (ZL): It refers to the above two groups (ZLJ and ZLBC); Zhangliang is the name of a township in Lushan city, Henan province, China;
